# Immunosenescence as a driver of the transition from frailty to multimorbidity

**DOI:** 10.3389/fimmu.2026.1810241

**Published:** 2026-05-21

**Authors:** Ruxia Qiu, Dong Zhang, Hui Feng

**Affiliations:** 1 Xiangya School of Nursing, Central South University, Changsha, China; 2 Ministry of Education Key Laboratory of Cell Proliferation and Regulation Biology, Beijing Key Laboratory of Gene Resource and Molecular Development, College of Life Sciences, Beijing Normal University, Beijing, China

**Keywords:** frailty, immunosenescence, inflammaging, multimorbidity, resilience

## Abstract

Frailty and multimorbidity are closely intertwined syndromes of later life, yet they are still commonly interpreted through parallel clinical frameworks rather than a shared biological mechanism. In this mini review, we propose that immunosenescence provides a unifying axis linking the transition from frailty to multimorbidity. Rather than representing a simple decline in immune function, immunosenescence is better understood as a maladaptive remodeling process characterized by constrained adaptive immune renewal, repertoire narrowing, chronic low-grade inflammation, impaired immune surveillance, and defective resolution and repair. We argue that these changes erode physiological reserve through convergent effects on skeletal muscle maintenance and regeneration, metabolic flexibility, neuroendocrine stress adaptation, and recovery after physiological perturbation, thereby promoting the emergence of frailty. The same immune alterations may then lower the threshold for parallel tissue-specific injury across cardiovascular, metabolic, neural, skeletal, and other systems, favoring non-random disease clustering and the development of multimorbidity. Once multimorbidity is established, disease-derived inflammatory and metabolic stressors may further accelerate immune dysregulation, creating a self-reinforcing cycle of vulnerability. We also highlight translational implications of this framework, including the need to move beyond single inflammatory markers toward integrated immune-ageing signatures and to test pathway-aligned interventions such as lifestyle optimization, vaccination strategies, immune tuning, and selected senescence-targeting approaches. A mechanistically grounded immunosenescence framework may help reorient late-life care toward preserving resilience and slowing chronic disease accumulation.

## 1 Introduction

Population ageing is increasing the burden of complex, disability-driving syndromes that shape late-life health trajectories. Two of the most consequential are frailty, a clinically recognizable state of diminished physiological reserve and heightened vulnerability to stressors, and multimorbidity, commonly defined as the coexistence of 2 or more chronic conditions within an individual (1, 2). These syndromes frequently co-occur and reinforce one another, accelerating disability, dependency, hospitalization, and mortality. Yet they are still commonly approached through parallel clinical and research frameworks. However, although frailty and multimorbidity are tightly linked epidemiologically, their biological relationship remains incompletely resolved; systematic evidence supports substantial overlap and possible bidirectionality, but not a sufficiently integrated mechanistic explanation of why frailty often precedes, accompanies, or amplifies multimorbidity (3, 4).

A plausible upstream axis is age-related immune remodeling. Broadly, immune ageing refers to cumulative changes in immune composition, function, and regulation across the lifespan. Within this broader process, immunosenescence is best understood not simply as uniform immune decline, but as a maladaptive remodeling characterized by constrained adaptive immune renewal, repertoire narrowing, altered effector differentiation, impaired immunosurveillance, and defective resolution and repair. Importantly, immune ageing may also coexist with compensatory adaptations that preserve selected aspects of host defense under lifelong antigenic exposure, although these adaptations may come at the cost of reduced repertoire flexibility and heightened inflammatory tone (5, 6). This process is closely intertwined with inflammaging, the chronic low-grade inflammatory state originally conceptualized by Franceschi and colleagues (7). It is also shaped by the senescence-associated secretory phenotype (SASP), a pro-inflammatory and tissue-remodeling secretome released by senescent cells that includes cytokines, chemokines, growth factors, and proteases (8). Together, these processes are better understood as remodeling rather than uniform immune decline. In humans, this remodeling is grounded in identifiable biological changes: maintenance of the adult naïve T-cell pool depends substantially on peripheral homeostatic proliferation rather than on sustained thymic output alone; age-associated repertoire attrition can create functional “holes” in antigen recognition; and chronic viral antigenic pressure, particularly from cytomegalovirus, can drive oligoclonal CD8+ T-cell expansions with highly differentiated CD28−CD57+CCR7− phenotypes (6, 9, 10). Recent multi-omic profiling further indicates that immune ageing is heterogeneous and non-linear, involving coordinated shifts in immune-cell composition and state that are not reducible to a few circulating cytokines (11).

These observations make immunosenescence a biologically plausible bridge between frailty and multimorbidity. On the one hand, maladaptive immune remodeling can recalibrate inflammatory set-points, weaken tissue surveillance, and impair resolution of injury, thereby eroding physiological resilience and lowering tolerance to stressors. On the other hand, the same immune disturbances may facilitate parallel pathology across multiple organ systems, increasing susceptibility to non-random disease clustering rather than isolated single-organ failure. Importantly, emerging human studies already suggest that immunosenescence-related immune phenotypes are linked to frailty, including CD28 loss and late-differentiated T-cell features (12, 13), whereas investigations in very old adults and longitudinal aging cohorts have begun to examine whether lymphocyte senescence profiles and leukocyte-derived indices are related to multimorbidity burden and mortality risk (14, 15). Yet these lines of evidence remain fragmented: frailty is still often discussed through inflammatory biomarkers alone, multimorbidity through disease-count epidemiology, and immunosenescence through isolated immune traits rather than an integrated cross-syndrome framework.

**Figure 1:**
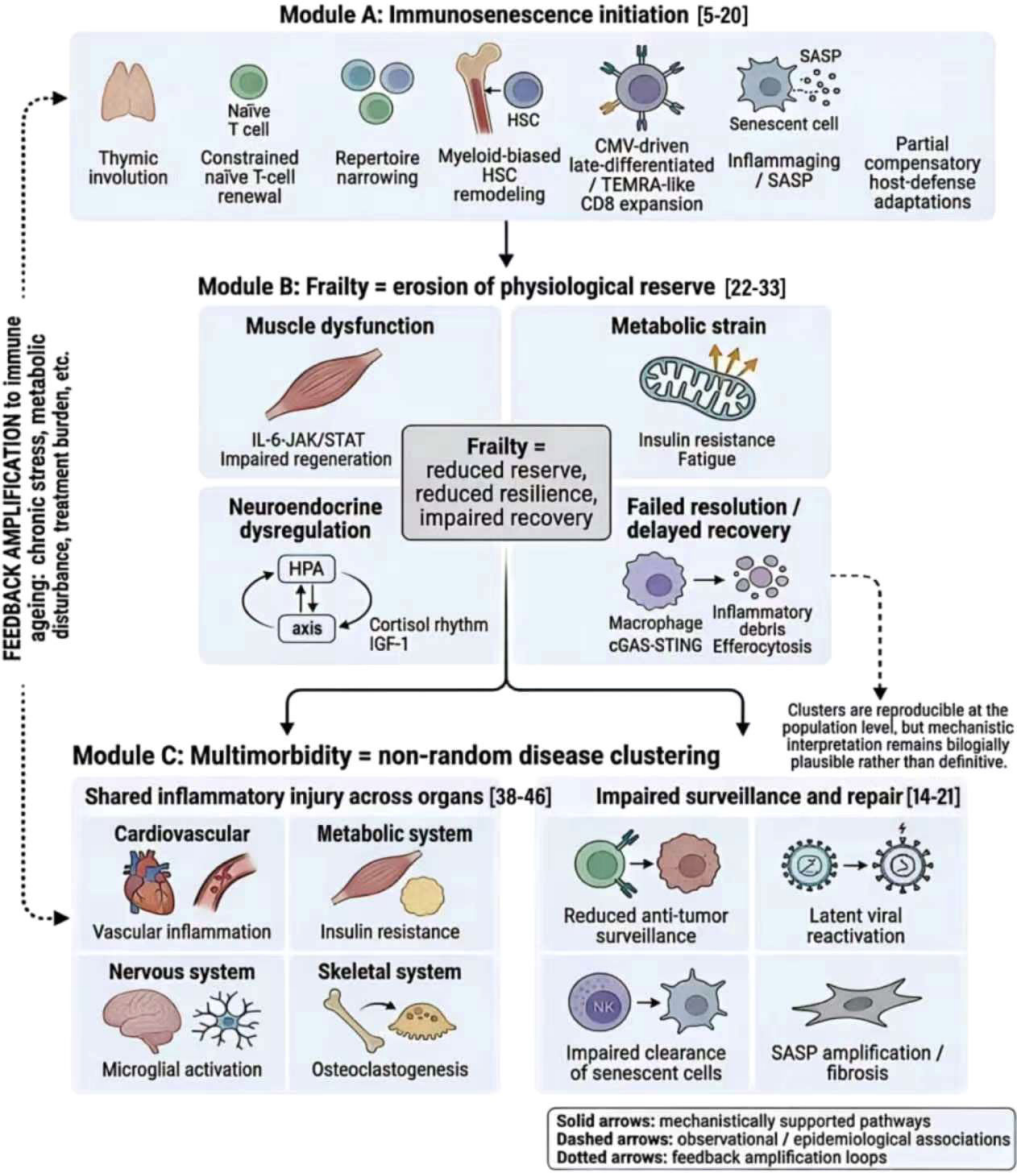
Mechanistic continuum linking immunosenescence, frailty, and multimorbidity.

Here, we propose that immunosenescence functions as a biological axis that helps explain the transition from frailty to multimorbidity. Specifically, we argue that age-related immune remodeling, through constrained adaptive renewal, persistent inflammatory signaling, impaired surveillance, and defective repair, contributes first to the erosion of physiological reserve that manifests clinically as frailty, and then to the accumulation and clustering of chronic diseases that define multimorbidity. Once multimorbidity is established, disease-derived inflammatory and metabolic stressors may in turn accelerate immune dysregulation, creating a self-reinforcing cycle. This proposed mechanistic continuum is summarized in **Figure 1**. In this mini review, we therefore synthesize classical and emerging evidence to frame frailty and multimorbidity not as parallel consequences of ageing alone, but as interlinked clinical expressions of a common immunobiological trajectory, and we outline pathway-aligned translational opportunities for preserving resilience in later life.

This figure summarizes the proposed continuum by which immunosenescence may contribute to the transition from frailty to multimorbidity. **Module A** depicts key features of immunosenescence initiation, including thymic involution, constrained naïve T-cell renewal, repertoire narrowing, myeloid-biased hematopoietic stem-cell remodeling, CMV-driven expansion of late-differentiated/TEMRA-like CD8+ T cells, and inflammaging/SASP, together with partial compensatory host-defense adaptations that may preserve selected functions at the cost of reduced immune flexibility. **Module B** illustrates frailty as erosion of physiological reserve, in which immune ageing converges on four major domains: muscle dysfunction, metabolic strain, neuroendocrine dysregulation, and failed resolution or delayed recovery. **Module C** shows how these processes may lower the threshold for multimorbidity through two broad pathways: shared inflammatory injury across organ systems and impaired surveillance and repair, thereby promoting non-random disease clustering. The outer loop indicates that once multimorbidity is established, chronic inflammatory and metabolic stress, together with treatment burden and related disturbances, may further amplify immune dysregulation and reinforce immunosenescence. Solid arrows indicate mechanistically supported pathways; dashed arrows indicate observational or epidemiological associations; dotted arrows indicate feedback amplification loops. Disease clusters are reproducible at the population level, whereas their mechanistic interpretation should be regarded as biologically plausible rather than definitive.

## 2 Immunosenescence initiation

Immunosenescence begins with age-related disruption of the systems responsible for adaptive immune renewal rather than with a simple, uniform decline in all immune functions. A central early change is thymic involution, which progressively constrains *de novo* naïve T-cell production. In humans, however, maintenance of the peripheral naïve T-cell pool depends substantially on post-thymic homeostatic proliferation rather than on sustained thymic output alone (5, 6). The consequence is therefore not merely numerical decline, but progressively restricted adaptive renewal and erosion of repertoire breadth, with the potential emergence of functional “holes” in antigen recognition (9). In parallel, ageing remodels the hematopoietic stem-cell compartment. Aged human and murine HSCs show reduced regenerative fitness and increasing myeloid-biased differentiation, with diminished lymphoid output and impaired support for adaptive immune replenishment (16, 17). Recent mechanistic work further suggests that this bias is not simply descriptive but actively driven by age-associated molecular programs, including clusterin-linked mitochondrial dysregulation (18). Together, thymic involution, constrained naïve-cell renewal, and myeloid-skewed hematopoiesis establish a cellular substrate for immunosenescence by limiting the renewal and diversity of lymphocyte populations.

These trajectories are further shaped by chronic antigenic stimulation and a persistent inflammatory tissue environment. Inflammaging, sustained by immune and stromal sources together with age-related tissue senescence, reinforces age-related shifts in both innate and adaptive immune states (7). In the T-cell compartment, ageing is associated with progressive enrichment of highly differentiated phenotypes marked by loss of CD28 and gain of CD57, features consistent with replicative constraint and reduced proliferative flexibility (19, 20). Chronic viral infection, particularly cytomegalovirus (CMV), is a major amplifier of this process. In older adults, CMV seropositivity drives oligoclonal expansion of virus-specific CD8+ T cells and favors the accumulation of highly differentiated CD8+ T-cell populations, consistent with later-described TEMRA enrichment (10). CMV-associated chronic antigenic stimulation preferentially promotes expansion of late effector-memory and TEMRA-like CD8+ T-cell compartments, thereby further constraining repertoire flexibility. As adaptive renewal becomes constrained and terminally differentiated clones expand, immune-cell composition shifts toward a state that is less diverse, less flexible, and more prone to sustaining inflammatory tone. Collectively, the convergence of impaired lymphoid renewal, myeloid skewing, chronic antigenic burden, and persistent low-grade inflammation initiates immunosenescence as a systems-level remodeling process that progressively erodes physiological reserve and resilience, thereby priming vulnerability to frailty and multimorbidity.

## 3 From immunosenescence to frailty: erosion of reserve

Immunosenescence may contribute to frailty not through a single inflammatory mediator, but through convergent impairment of systems that determine reserve and recovery. In this framework, maladaptive immune remodeling lowers resilience by disrupting muscle maintenance and regeneration, reducing metabolic flexibility, altering neuroendocrine stress adaptation, and slowing recovery after physiological perturbation (**Figure 1**). Frailty then emerges when ordinarily manageable stressors produce disproportionate and incompletely reversible losses in strength, mobility, endurance, and function, rather than being fully compensated by intact multisystem reserve (1, 21).

### 3.1 Muscle as a downstream effector: inflammatory signaling, anabolic resistance, and impaired regeneration

Skeletal muscle is a major downstream organ through which immune ageing becomes clinically visible. Chronic low-grade inflammatory signaling is not specific to immunosenescence, but it can engage catabolic pathways that are highly relevant to muscle decline. Experimental work shows that IL-6/gp130/JAK2/STAT3 signaling can directly drive muscle wasting, while longitudinal human data indicate that higher IL-6 and CRP are associated with subsequent loss of appendicular skeletal muscle mass. These observations support the view that anabolic resistance is not only a nutritional phenomenon, but also a downstream consequence of a chronically dysregulated immune-metabolic environment (22, 23).

Beyond circulating mediators, immune ageing also remodels the local regenerative niche. Senescent cells are integral components of the skeletal-muscle repair environment and can repress regeneration, while ageing alters the expansion and inflammatory resolution programs of niche macrophages, thereby impairing muscle stem-cell support (24, 25). Ageing is also accompanied by deficient local biosynthesis of specialized pro-resolving mediators in muscle, further favoring maladaptive remodeling over efficient repair (26). More recently, bioactive lipid class switching has been shown to regulate myogenesis and muscle regeneration, and exogenous Protectin-D1 restored myogenesis and improved regeneration in preclinical models, reinforcing the idea that failed resolution is mechanistically important rather than epiphenomenal (27). Over time, these convergent defects favor sarcopenic decline, especially losses of strength and physical performance, which overlap directly with the physical phenotype of frailty (1).

### 3.2 Metabolic inefficiency: immune-metabolic strain, insulin resistance, and fatigue

Metabolic inefficiency represents a second route through which immune ageing may erode reserve. Sustained immune activation is energetically costly and can reduce metabolic flexibility, especially when older adults encounter superimposed stressors such as illness, immobilization, or undernutrition. A key mechanistic point is that inflammatory signaling can directly impair insulin action in skeletal muscle: in healthy humans, TNF-α infusion induces skeletal-muscle insulin resistance without affecting endogenous glucose production, providing causal evidence that inflammatory perturbation can reduce metabolic efficiency (28). Because skeletal muscle is the dominant site of postprandial glucose disposal, this shift has consequences beyond glycemia, including greater fatigability, lower endurance, and reduced capacity to sustain activity. These clinical consequences map closely onto exhaustion and low physical activity, which are central dimensions of frailty (1).

### 3.3 Neuroendocrine reserve: altered HPA dynamics and reduced anabolic responsiveness

Neuroendocrine reserve provides a third pathway linking immune ageing to frailty. In community-based studies, frailty has been associated with altered diurnal cortisol dynamics, including higher cortisol burden and a blunted daily rhythm, findings consistent with hypothalamic-pituitary-adrenal axis dysregulation (29, 30). Such patterns may weaken temporal control of inflammation and bias the organism toward inefficient stress responses. In parallel, lower circulating IGF-1 has been associated with frailty in older adults, supporting the idea that reduced anabolic responsiveness and neuroendocrine dysregulation may converge biologically (31). Because these data are largely observational, they should be interpreted as supportive rather than definitive; however, they are consistent with a model in which inflammatory tone and endocrine vulnerability jointly reduce reserve and impair recovery from stress.

### 3.4 Recovery failure: impaired resolution as a mechanistic bridge to vulnerability

A defining feature of frailty is not simply baseline deficit, but impaired recovery after perturbation. Immune ageing can contribute to this through reduced immune protection, constrained metabolic and endocrine flexibility, and defective resolution of tissue injury. Of these mechanisms, impaired resolution is especially informative because it connects immune dysfunction directly to functional recovery. With ageing, macrophage efferocytosis becomes less efficient; this delays clearance of dying cells and inflammatory debris and can aberrantly activate innate sensing pathways such as cGAS-STING, thereby sustaining type I interferon programs and delaying restoration of tissue homeostasis (32). Recent work has further shown that sterile tissue injury can generate spatially organized type I interferon responses at injury borderzones, supporting the broader plausibility that persistent innate immune signaling may prolong tissue dysfunction after injury (33). Clinically, the implication is that older individuals with immunosenescent biology may be less able to return promptly to baseline function after infection, minor injury, or hospitalization, making recovery kinetics, and not only static deficits, a key expression of frailty.

### 3.5 Evidence linkage: from single inflammatory markers to immune phenotypes and composite clocks

Evidence linking immune ageing to frailty should not be reduced to IL-6 and CRP alone. Original cohort studies have shown that higher IL-6 and inflammatory burden are associated with prevalent frailty and with adverse outcomes in frail older adults (34, 35). However, single soluble markers remain biologically nonspecific: they may reflect adiposity, multimorbidity, acute illness, or broader inflammatory load rather than immunosenescence per se. Their value therefore lies more in indicating inflammatory burden than in defining an immune-ageing endotype.

A more informative bridge comes from studies that move beyond soluble analytes alone. In human cohorts, physical frailty has been linked to T-cell senescence phenotypes, particularly loss of CD28 expression and other late-differentiated T-cell features, while immunosenescence-associated T-cell phenotypes have also predicted frailty and short-term mortality in nursing-home residents (12, 13). At the integrative level, the inflammatory ageing clock iAge was developed to capture systems-level inflammatory ageing and was reported to track multimorbidity, immunosenescence, frailty, and cardiovascular ageing (36). More recently, a composite inflammaging score based on IL-6, IL-10, and CXCL9 was associated with frailty and long-term mortality in hospitalized older adults (37), and multi-omic immune profiling has shown that immune ageing is heterogeneous, cell-state dependent, and not reducible to a small set of circulating cytokines (11). Together, these findings suggest that frailty-relevant immune biology is better captured by multivariable signatures that integrate cellular and soluble dimensions than by any single biomarker alone. For clinical translation, the key next step is to determine whether such immune-ageing signatures improve prediction of incident frailty or post-stressor recovery beyond age, comorbidity burden, and baseline frailty severity, and whether they are responsive to intervention.

## 4 From frailty to multimorbidity: immune-mediated multi-organ vulnerability and disease clustering

Frailty may progress to multimorbidity when impaired reserve is repeatedly converted into organ-specific pathology across tissues that share vulnerability to chronic inflammation, defective immune surveillance, and impaired repair (**Figure 1**). In this framework, immunosenescence does not imply that all chronic diseases arise from a single mechanism; rather, it provides a shared biological background that lowers the threshold for dysfunction in multiple organs simultaneously. Once multimorbidity is established, disease-specific inflammatory and metabolic stressors may in turn further accelerate immune ageing, creating a self-reinforcing cycle of vulnerability, disease accumulation, and functional decline.

### 4.1 Systemic inflammation as a shared driver of multi-organ pathophysiology

A major route through which frailty may transition toward multimorbidity is the spread of immune-mediated injury across organ systems. This is not simply a matter of elevated cytokine levels, but of shared inflammatory programs being translated into tissue-specific pathology.

(a) Cardiovascular system.

The vasculature is one of the clearest targets of chronic inflammatory signaling. In stable coronary heart disease, higher IL-6 levels independently predict major adverse cardiovascular events, cardiovascular mortality, myocardial infarction, heart failure, and even cancer mortality, suggesting that upstream inflammatory tone has pleiotropic relevance across outcomes rather than organ-restricted effects alone (38). Clinical proof-of-principle also comes from CANTOS, in which IL-1β inhibition with canakinumab reduced recurrent cardiovascular events without lowering lipid levels, supporting a causal role for inflammatory signaling in atherothrombotic disease progression (39).

(b) Metabolic system.

Inflammatory signaling can also generate parallel metabolic vulnerability. In healthy humans, TNF-α infusion induces skeletal-muscle insulin resistance without affecting endogenous glucose production, providing direct experimental evidence that inflammatory perturbation can impair peripheral glucose handling (28). This mechanism helps explain why cardiometabolic diseases frequently cluster rather than occur in isolation, particularly when frailty has already reduced metabolic reserve.

(c) Nervous system.

The nervous system is similarly exposed to the consequences of peripheral inflammatory disturbance. Multiomic work has shown that blood components can induce broad microglial transcriptional changes involving oxidative stress, type I interferon signaling, and neurodegenerative programs, and that fibrinogen is one key driver of these blood-induced microglial responses (40). Thus, age-related failure to contain peripheral inflammation may promote central neuroimmune activation and contribute to cognitive and neurodegenerative vulnerability.

(d) Skeletal system.

Bone is another tissue in which inflammatory pathways can be converted into structural damage. TNF-α markedly enhances osteoclastogenesis when osteoclast precursors are primed by otherwise subthreshold levels of RANKL, indicating that inflammatory signals can amplify bone resorption rather than merely accompany it (41). In addition, IL-6 supports osteoclast development through osteoblast-lineage signaling, providing a mechanistic link between chronic inflammatory tone and remodeling imbalance (42). These pathways offer a biologically plausible route by which frailty-associated inflammatory vulnerability may facilitate osteoporosis and fracture-prone multimorbidity.

### 4.2 Consequences of reduced immune surveillance and impaired repair capacity

Multimorbidity cannot be explained by chronic inflammatory signaling alone. Immunosenescence also involves declining capacity to detect, contain, and repair tissue-level damage. This becomes clinically relevant when failures of surveillance allow malignant, infected, or senescent cells to persist and reshape the surrounding tissue environment.

(a) Increased cancer susceptibility.

Preclinical evidence suggests that ageing compromises tumor control not only by increasing inflammatory tone, but also by directly impairing adaptive antitumor immunity. In an aged tumor microenvironment, antigen-specific CD8+ T cells show reduced persistence and proliferation, and tumor control is weakened relative to younger hosts (43). These data support the broader concept that immunosenescence can facilitate cancer emergence and progression by diminishing effective immune surveillance.

(b) Latent viral reactivation.

A clinically relevant example of surveillance failure is the reactivation of latent varicella-zoster virus. Recent work shows that ageing is associated with reduced VZV-specific cell-mediated immunity together with altered latent viral load dynamics, reinforcing the view that weakened antigen-specific immune control contributes to herpes zoster susceptibility in older adults (44). Such episodic reactivation adds acute morbidity to an already growing chronic disease burden.

(c) Impaired clearance of senescent cells.

Senescent-cell accumulation is also shaped by immune failure. In human senescent fibroblasts, HLA-E upregulation can engage the inhibitory receptor NKG2A on NK cells and highly differentiated CD8+ T cells, thereby protecting senescent cells from immune-mediated elimination (45). More recently, experimental live-cell models have shown that NK cells can directly clear senescent renal tubular epithelial cells, underscoring that immune removal of senescent cells is a real biological process, not only a theoretical construct (46). With immune ageing, diminished clearance may allow senescent cells to accumulate, sustain SASP signaling, and amplify fibrosis, inflammation, and regenerative failure across tissues.

### 4.3 Interpreting reproducible multimorbidity patterns and disease clustering

Large population-based analyses increasingly show that multiple long-term conditions organize into reproducible clusters rather than accumulating randomly (47, 48). These clusters often involve recurring cardiometabolic, musculoskeletal, neuropsychiatric, renal, and cancer-related combinations, suggesting that shared upstream drivers operate across tissues. A mechanistic interpretation is that immune ageing contributes to this patterning by coupling chronic inflammation, altered innate immune activation, and impaired adaptive surveillance to organ systems that differ in phenotype but overlap in underlying vulnerability.

Importantly, this interpretation should be framed as a biologically plausible explanation rather than a proven sole cause of multimorbidity clustering. Markers such as IL-6 and CRP are associated with risk across several disease domains, but they remain nonspecific and may reflect broader disease activity or comorbidity burden rather than immunosenescence itself (38). Even so, when the same inflammatory and surveillance failures can plausibly drive endothelial dysfunction, insulin resistance, neuroimmune activation, and impaired bone remodeling, it becomes easier to understand why frailty may evolve into non-random disease clustering rather than isolated single-organ pathology.

### 4.4 Multimorbidity as an amplifier of immune ageing processes

Once multimorbidity is established, chronic disease states can further intensify immune dysregulation and feed back into the immunosenescence process. Chronic kidney disease is a particularly important example: in patients on hemodialysis, higher levels of protein-bound uremic toxins are associated with reductions in less differentiated T- and B-cell subsets and with more immunosenescent and immunoexhausted lymphocyte profiles, indicating that disease-related metabolic products can actively reshape immune ageing phenotypes (49). This supports the idea that multimorbidity is not just a downstream consequence of immune ageing, but can also become an upstream accelerator of it.

Polypharmacy adds a further layer of complexity. In older adults with multimorbidity, inflammatory profiles associated with polypharmacy were not reproduced in healthy aged mice receiving multiple medications, suggesting that medication burden may interact with underlying disease biology rather than acting independently of it (50). This finding is important because it implies that multimorbidity-related immune heterogeneity may arise from the combined effects of chronic disease, treatment exposure, and ageing biology, rather than from any single factor alone. Collectively, these processes support a self-perpetuating cycle in which frailty predisposes to multimorbidity, multimorbidity amplifies immune dysfunction, and progressive immune dysregulation further accelerates disease accumulation and functional decline. Key immune-ageing alterations linking frailty and multimorbidity are summarized in **Table 1**, together with representative markers, putative mechanisms, and candidate interventions.

**Table 1:** Summary of immune-ageing alterations linking frailty and multimorbidity and their translational implications.

Immune-ageing alteration	Representative cells, markers, or signatures	Frailty link	Multimorbidity link	Putative mechanism	Candidate interventions
Constrained adaptive immune renewal	Thymic involution; reduced *de novo* naïve T-cell output; reduced naïve CD4+/CD8+ pools; repertoire narrowing; age-related “holes” in TCR diversity; myeloid-biased HSCs	Reduces physiological reserve and adaptive flexibility; may increase vulnerability to infection, poor recovery, and stress intolerance	Lowers the threshold for accumulation of multiple chronic conditions by weakening immune adaptability across tissues	Restricted adaptive renewal limits antigenic breadth, impairs immune responsiveness, and reduces resilience under repeated physiological stress	Protein-adequate nutrition; structured exercise; immune reconstitution strategies (investigational); vaccine optimization
Terminal T-cell differentiation and CMV-driven remodeling	CD28 loss; CD57 gain; late-differentiated CD8+ T cells; TEMRA enrichment; oligoclonal CD8+ expansion; CMV seropositivity	Linked to physical frailty and, in some cohorts, to short-term mortality and sarcopenic phenotypes	May contribute to chronic inflammatory tone, impaired surveillance, and broader multimorbidity vulnerability	Chronic antigenic stimulation drives clonal inflation, reduced proliferative flexibility, and skewed effector-memory dominance	Immune-phenotyping-guided risk stratification; vaccination strategies; immune-tuning approaches such as low-dose mTOR modulation (investigational in this context)
Inflammaging and SASP amplification	IL-6, CRP, TNF-α, IL-1β; SASP-associated cytokines and chemokines; composite inflammatory signatures (e.g., IL-6/IL-10/CXCL9-based scores)	Linked to exhaustion, low activity, sarcopenia, and broader frailty phenotypes	Promotes parallel injury across cardiovascular, metabolic, neural, and skeletal systems, favoring disease clustering	Chronic low-grade inflammatory signaling recalibrates systemic set-points and amplifies tissue-specific damage and remodeling	Exercise; nutritional optimization; selected anti-inflammatory pathway inhibition in carefully chosen populations; senescence-targeting approaches (investigational)
Macrophage dysfunction and failed resolution	Reduced efferocytosis; delayed inflammatory-to-pro-regenerative transition; deficient pro-resolving mediators; cGAS-STING and type I IFN signaling	Slows recovery after stressors; impairs muscle regeneration; contributes to stepwise functional decline	Sustains tissue injury, fibrosis, and chronic organ dysfunction, thereby facilitating persistence and accumulation of conditions	Defective clearance of dying cells and debris prolongs inflammatory signaling and biases tissue repair toward maladaptive remodeling	Exercise; nutritional support; resolution-targeted or pro-resolving strategies (investigational)
Impaired immune surveillance	Reduced NK-cell activity; exhausted/late-differentiated T-cell phenotypes; reduced antigen-specific memory responses; HLA-E/NKG2A-mediated escape of senescent cells	Increases susceptibility to infection-related decompensation and may worsen recovery trajectories	Facilitates cancer susceptibility, latent viral reactivation, and senescent-cell accumulation, thereby broadening disease burden	Weak control of malignant, infected, or senescent cells permits persistence of pathology and local inflammatory amplification	Optimized vaccination; investigational approaches to enhance NK- and T-cell surveillance; senescence-targeting approaches (investigational)
Immune-metabolic dysregulation	Inflammatory insulin resistance; reduced metabolic flexibility; fatigue/endurance-related phenotypes; altered glucose handling in skeletal muscle	Contributes to exhaustion, low physical activity, and reduced stress tolerance	Helps explain recurrent cardiometabolic clustering, especially diabetes–cardiovascular combinations	Sustained immune activation increases energetic cost and impairs insulin signaling, thereby reducing reserve and promoting metabolic inefficiency	Exercise; protein and energy adequacy; cardiometabolic risk-factor control; immunometabolic modulation (investigational)
Neuroendocrine dysregulation	Blunted diurnal cortisol rhythm; higher cortisol burden; lower IGF-1; altered anabolic responsiveness	Reduces neuroendocrine reserve and impairs recovery from physical or inflammatory stress	May amplify cross-system vulnerability and increase the likelihood that acute stressors precipitate chronic disease progression	Dysregulated HPA-axis signaling and reduced anabolic responsiveness weaken temporal control of inflammation and tissue maintenance	Structured exercise; nutritional support; optimization of comorbidity management; mechanism-targeted endocrine/immune approaches remain exploratory
Composite immune-ageing signatures	iAge; IL-6/IL-10/CXCL9 inflammaging score; leukocyte-derived indices (for example, NLR); multi-omic immune profiles	May improve frailty risk stratification beyond single cytokines	May help identify individuals at higher risk of multimorbidity burden, progression, and mortality	Integrates cellular and soluble immune features into systems-level immune-ageing phenotypes	Biomarker-guided enrichment and stratification in longitudinal studies and pathway-aligned intervention trials

Abbreviations: CMV, cytomegalovirus; HPA, hypothalamic-pituitary-adrenal; HSC, hematopoietic stem cell; IFN, interferon; NLR, neutrophil-to-lymphocyte ratio; NK, natural killer; SASP, senescence-associated secretory phenotype; TCR, T-cell receptor; TEMRA, terminally differentiated effector memory T cells re-expressing CD45RA.

This table summarizes major immune-ageing alterations discussed in this review and highlights their putative links to frailty, multimorbidity, and translational intervention opportunities. The proposed mechanisms are intended as an integrative conceptual framework rather than definitive proof of causality for every pathway. Candidate interventions differ substantially in evidentiary maturity; lifestyle strategies and vaccine optimization are currently the most clinically applicable, whereas immune reconstitution, senolytics, and several immune-tuning approaches remain investigational.

## 5 Clinical and translational implications

A more useful translational approach is to align interventions with the principal biological deficits highlighted in this review, including persistent inflammatory signaling, impaired resolution and tissue repair, reduced adaptive immune renewal, and increased susceptibility to infection-related decompensation. On this basis, lifestyle strategies remain foundational, pharmacologic immune-tuning may be justified in selected high-risk individuals, and immune reconstitution is best viewed as an emerging investigational direction rather than routine care.

### 5.1 Lifestyle interventions

Lifestyle interventions remain the most pragmatic approach for mitigating immunosenescence-related vulnerability as frailty progresses toward multimorbidity. In practice, nutritional management should first correct energy and protein inadequacy and address clinically relevant micronutrient deficiency, because low reserve magnifies the physiological consequences of undernutrition. For most older adults, protein targets around 1.0–1.2 g/kg/day are reasonable, with higher intakes considered during illness or rehabilitation but individualized to renal function and metabolic tolerance (51). In parallel, structured aerobic plus resistance exercise is the most plausible nonpharmacologic immune-tuning strategy and has been associated with a higher proportion of naïve T lymphocytes and a more favorable CD4/CD8 balance in older adult (52). Microbiome-directed interventions may serve as adjuncts, but current evidence remains modest and strain-specific rather than sufficient for stand-alone use (53). Overall, the goal is not simply to reduce inflammation, but to preserve reserve and reduce the likelihood that everyday stressors precipitate functional decline.

### 5.2 Pharmacologic interventions

Pharmacologic strategies can be grouped according to the lesion they are intended to modify: senescence-linked processes, inflammatory hub pathways, and immunometabolic regulation. Senolytics currently remain early translational candidates; for example, a first-in-human pilot study of dasatinib plus quercetin in idiopathic pulmonary fibrosis suggested feasibility and possible functional benefit, but the evidence remains small, uncontrolled, and disease-specific (54). Targeting inflammatory hub pathways is supported by proof-of-concept from CANTOS, in which IL-1β inhibition reduced recurrent cardiovascular events but increased infection-related adverse events, underscoring the importance of careful subgroup selection in older adults with frailty or multimorbidity (39). Low-dose mTOR/TORC1 inhibition provides the clearest current example of immune tuning rather than immune suppression, having improved influenza vaccine responses and reduced infection burden in older adults in early trials (55, 56). By contrast, NAD^+^-augmenting approaches remain investigational: although NMN and nicotinamide riboside can raise systemic NAD^+^ and may influence selected functional outcomes, reproducible benefits on immunosenescence-relevant endpoints remain unproven (57–60).

### 5.3 Immune reconstitution

Immune reconstitution is biologically attractive only when the target lesion is clearly specified. In the present framework, it is most relevant to loss of adaptive renewal, particularly thymic involution and progressive restriction of *de novo* naïve T-cell production. The main human proof-of-concept comes from the TRIIM study, in which a small uncontrolled intervention combining growth hormone, metformin, and DHEA was associated with favorable changes in thymic imaging, immune-cell measures, and epigenetic-age readouts (61). However, the evidence remains preliminary because of limited sample size, lack of a conventional control group, and short follow-up. At present, immune reconstitution should therefore be regarded as an investigational strategy rather than an established option for routine multimorbidity care.

### 5.4 Optimizing vaccination strategies

For older adults with frailty or multimorbidity, vaccination should be considered a practical strategy to reduce infection-triggered decompensation rather than a stand-alone preventive measure. Existing data already show that age-related immune constraints can be partly overcome by more immunogenic platforms: high-dose influenza vaccination was more efficacious than standard-dose vaccination in adults aged 65 years or older, and recombinant zoster vaccination substantially reduced herpes zoster and postherpetic neuralgia in adults aged 70 years or older (62, 63). An important translational question is whether vaccine protection can be further strengthened through short-term immune tuning. Early trials of mTOR/TORC1 inhibition suggest that this is biologically plausible, as brief modulation improved vaccine immunogenicity and was also associated with reduced infection burden in older adults (55, 56). In practice, vaccination is most likely to be effective when integrated with broader measures that support immune resilience, including nutritional optimization, structured exercise, and tighter control of coexisting chronic conditions. The next priority is to determine whether such mechanism-informed strategies can reduce downstream outcomes that matter most in frail and multimorbid populations, particularly serious infections, hospitalization, and functional deterioration.

## 6 Conclusion

Frailty and multimorbidity are closely intertwined geriatric syndromes, yet they are still commonly interpreted through organ-specific frameworks. In this review, we argue that immunosenescence provides a mechanistic basis for understanding how frailty may progress toward multimorbidity. Through the maladaptive convergence of immune remodeling and inflammaging, immune ageing can recalibrate inflammatory set-points, constrain adaptive immune renewal, weaken immune surveillance, and impair tissue repair, thereby eroding musculoskeletal, metabolic, and neuroendocrine reserve and increasing susceptibility to parallel tissue-specific injury and non-random disease clustering. Once multimorbidity is established, disease-derived inflammatory and metabolic stressors, together with treatment burden and polypharmacy, may further amplify immune dysregulation, creating a self-reinforcing cycle of vulnerability and chronic disease accumulation. A key translational priority is therefore to move beyond single inflammatory markers toward integrated immunosenescence signatures that capture immune-cell phenotypes, soluble mediators, and multi-omic features, and that improve prediction of recovery trajectories and multimorbidity risk beyond chronological age alone. Future progress will depend on longitudinal, stressor-anchored studies and pathway-aligned intervention trials testing scalable lifestyle strategies, optimized vaccination, and carefully risk-stratified immune-modulating or senescence-targeting approaches. A more mechanistic understanding of immunosenescence may help reframe late-life care toward preserving resilience and slowing chronic disease accumulation.
