# Correction: Immunosenescence as a driver of the transition from frailty to multimorbidity

**DOI:** 10.3389/fimmu.2026.1887913

**Published:** 2026-05-28

**Authors:** Ruxia Qiu, Dong Zhang, Hui Feng

**Affiliations:** 1 Xiangya School of Nursing, Central South University, Changsha, China; 2 Ministry of Education Key Laboratory of Cell Proliferation and Regulation Biology, Beijing Key Laboratory of Gene Resource and Molecular Development, College of Life Sciences, Beijing Normal University, Beijing, China

**Keywords:** frailty, immunosenescence, inflammaging, multimorbidity, resilience

An incorrect **Funding** statement was provided. The **Funding** statement was published as:

“This work was supported by the Key Research and Development Program of Hunan Province (2025JK2118).”

The correct **Funding** statement reads:

“This study was supported by grants from the National Natural Science Foundation of China (Grant Nos. 72374224 and 72174212 to Hui Feng).”

The original version of this article has been updated.

